# Diagnostic randomized controlled trials: the final frontier

**DOI:** 10.1186/1745-6215-13-137

**Published:** 2012-08-16

**Authors:** Marc Rodger, Tim Ramsay, Dean Fergusson

**Affiliations:** 1 Thrombosis Program, Division of Hematology, Department of Medicine, Faculty of Medicine, University of Ottawa, 501 Smyth Road, Ottawa, ON K1H 8L6, Canada; 2 Clinical Epidemiology Program, Ottawa Hospital Research Institute, 501 Smyth Road, Ottawa, ON K1H 8L6, Canada; 3 Department of Epidemiology and Community Medicine, Faculty of Medicine, University of Ottawa, 501 Smyth Road, Ottawa, ON K1H 8L6, Canada; 4 Ottawa Methods Centre, Ottawa Hospital Research Institute, 501 Smyth Road, Ottawa, ON K1H 8L6, Canada; 5 The Ottawa Hospital, Clinical Epidemiology Program, 501 Smyth Road, Box 201, Ottawa, ON K1H 8L6, Canada

**Keywords:** Clinical trials, diagnostic tests, randomization

## Abstract

Clinicians, patients, governments, third-party payers, and the public take for granted that diagnostic tests are accurate, safe and effective. However, we may be seriously misled if we are relying on robust study design to ensure accurate, safe, and effective diagnostic tests. Properly conducted, randomized controlled trials are the gold standard for assessing the effectiveness and safety of interventions, yet are rarely conducted in the assessment of diagnostic tests. Instead, diagnostic cohort studies are commonly performed to assess the characteristics of a diagnostic test including sensitivity and specificity. While diagnostic cohort studies can inform us about the relative accuracy of an experimental diagnostic intervention compared to a reference standard, they do not inform us about whether the differences in accuracy are clinically important, or the degree of clinical importance (in other words, the impact on patient outcomes). In this commentary we provide the advantages of the diagnostic randomized controlled trial and suggest a greater awareness and uptake in their conduct. Doing so will better ensure that patients are offered diagnostic procedures that will make a clinical difference.

## Background

Clinicians rely heavily on diagnostic procedures to decide whether patients have or do not have a given condition or disease. Clinicians, patients, governments, third-party payers, and the public take for granted that such diagnostic tests are accurate, safe and effective. Yet what is the evidence that they are accurate, safe, and effective? If robust study design is the yardstick, we may be seriously misled. Properly conducted, randomized controlled trials are the gold standard for assessing the effectiveness and safety of interventions. While the publication of randomized trials of non-therapeutic interventions such as surgical procedures and behavioral interventions lag far behind those of drugs in number, randomized trials of diagnostic procedures are an even rarer species. To our knowledge, reasons for their lack of conduct have not been explored but may include the resources, sample size, and interdisciplinary teamwork required. Moreover, regulatory approval does not require randomized trials in their decision making. In order to adequately assess the diagnostic characteristics, as well as the impact on clinical outcomes without bias, we suggest that the evaluation of diagnostic interventions move beyond traditional diagnostic study designs to diagnostic randomized controlled trials.

**Figure 1:**
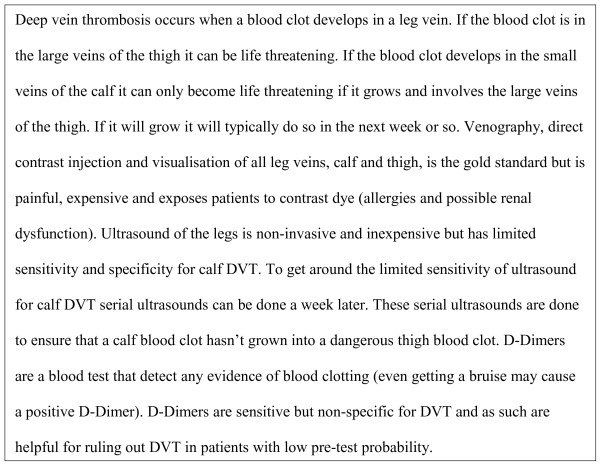
Primer on venous thrombosis.

To illustrate the need for diagnostic randomized controlled trials, consider the following scenario:

A patient presents to the emergency department with a painful swollen leg and the emergency room resident suspects deep vein thrombosis (DVT) (Figure1). The emergency room resident outlines a diagnostic management plan for the emergency room physician. He suggests a d-Dimer and a leg ultrasound be done simultaneously. The emergency room physicians points out that “if you are ordering an ultrasound why bother with ordering a D-Dimer if the patient has a low pre-test probability without diagnostic imaging?” The emergency resident pulls out a published diagnostic randomized controlled trial comparing a strategy of using D-Dimer after ultrasound (and venography if the D-Dimer is positive and ultrasound is negative) compared to serial ultrasounds alone without D-Dimer [1]. This diagnostic randomized controlled trial showed that the D-Dimer and ultrasound approach picked up 4% more DVTs than the serial ultrasound approach “so it is a superior approach as it is much more sensitive and specific”. The emergency room physician who also read the same paper points out that after follow-up there were no differences in important clinical outcomes between the two diagnostic strategies. Furthermore, the likely discrepancy is that the D-Dimer and ultrasound approach “picks up” more clinically insignificant calf blood clots. This example highlights important differences in diagnostic accuracy between cohort studies and diagnostic randomized controlled trials. The resident was interpreting the study by solely focusing on the diagnostic characteristics while ignoring clinically relevant outcomes.

## Main text

### The cohort design

**Figure 2:**
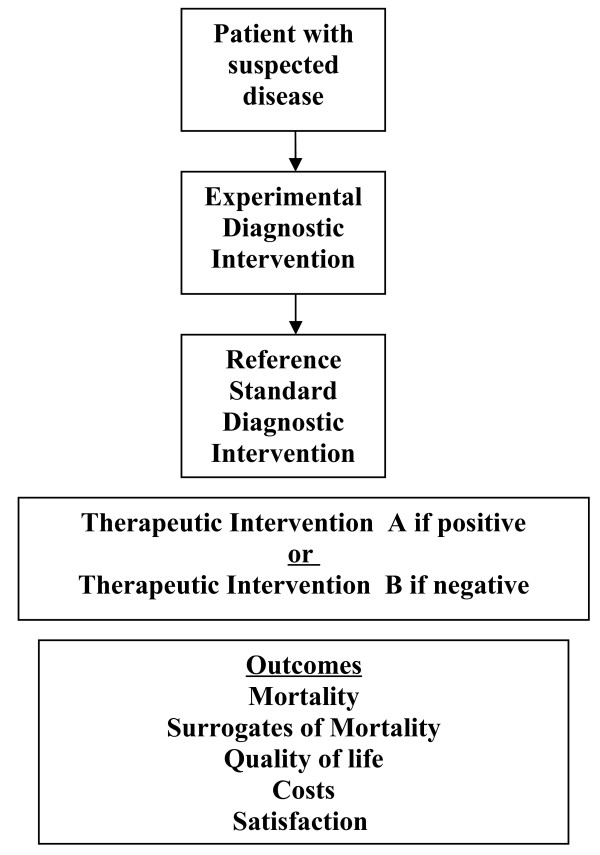
Prospective diagnostic accuracy cohort study.

The prevailing study design for investigating a new diagnostic test is a prospective blind comparison of the experimental test and the diagnostic reference standard (“gold standard”) in a consecutive series of patients from a representative clinical population [2-4]. In these prospective diagnostic accuracy cohort studies, patients with suspected disease undergo both an experimental diagnostic intervention and the diagnostic reference standard (Figure2). Diagnostic accuracy or the performance of the experimental diagnostic intervention is measured using a 2 × 2 table, and sensitivity, specificity, likelihood ratios, diagnostic odd ratios and accuracy can be calculated. Indeed, much literature has been published on the standards that should be met when evaluating diagnostic studies and this literature is primarily focused on diagnostic accuracy cohort studies [2-5]. The advantages of a diagnostic accuracy cohort study include their simplicity to perform, they are relatively inexpensive, and they are well accepted among the medical research community. They are appropriate for the early investigation of an experimental diagnostic test to determine if it is appropriate to continue investigating the experimental diagnostic test. However, while providing diagnostic accuracy information, diagnostic accuracy cohort studies are not directly tied to patient outcomes (Figure2). Indeed, we often forget that diagnostic tests alone do not improve patient outcomes. Only when diagnostic accuracy is coupled with effective therapy (or noxious therapy) can outcomes be influenced.

### The diagnostic randomized controlled trial

Twenty-five years ago, Guyatt and colleagues proposed that “diagnostic technology should be disseminated only if they are less expensive, produce fewer untoward effects, at least as accurate as existing technologies, eliminate the need for other diagnostic interventions, without loss of accuracy or lead to the institution of effective therapy” [6]. They further stated that “establishing patient benefit often requires randomized controlled trials”. This call to action continues to be advocated in the literature but largely ignored [7,8].

**Figure 3:**
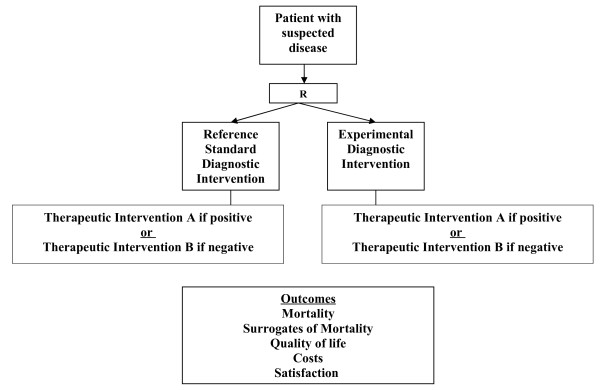
Open diagnostic intervention randomized controlled trial.

**Figure 4:**
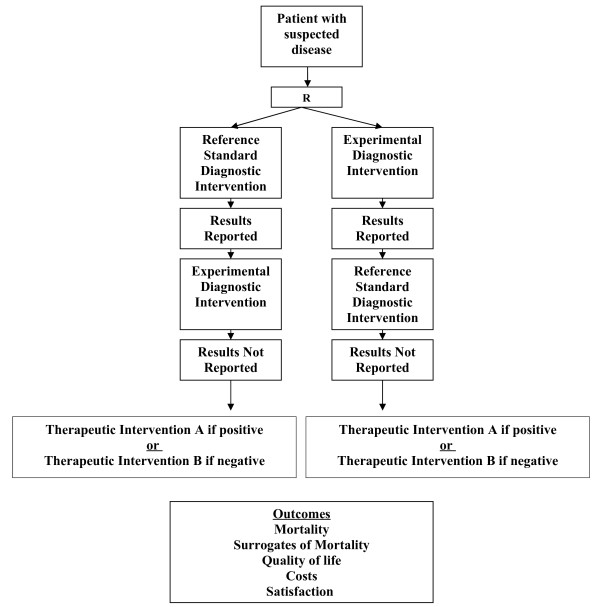
Blind diagnostic intervention randomized controlled trial.

We define diagnostic randomized controlled trials as randomized comparisons of two diagnostic interventions (one standard and one experimental) with identical therapeutic interventions based on the results of the competing diagnostic interventions (for example, disease: yes or no) and with the study outcomes being clinically important consequences of diagnostic accuracy (Figures 3 and 4). While diagnostic cohort studies inform us about the relative accuracy of an experimental diagnostic intervention compared to a reference standard, they do not inform us about whether the differences in accuracy are clinically important, or the degree of clinical importance (in other words, the impact on patient outcomes).

We propose that conducting diagnostic randomized controlled trials is critical in the evaluation of diagnostic technologies and, in particular, novel technologies in the presence of standard diagnostic tests. Our reasons include the following: 1) diagnostic randomized controlled trials permit a direct comparison of the experimental test to the standard test with clinically relevant outcomes as opposed to simply comparing them to each other; 2) diagnostic randomized controlled trials can result in the experimental test being better than the reference standard (which by definition cannot occur with diagnostic cohort studies); 3) diagnostic randomized controlled trials can be conducted where there is no accepted reference standard (which cannot be done with diagnostic cohort studies); 4) test properties (sensitivity, specificity, likelihood ratios, accuracy etc.) can still be calculated in diagnostic randomized controlled trials if the reference standard is conducted and the results kept blinded in the experimental group and the results of the experimental tests are conducted but kept blinded in the reference group; and 5) diagnostic technologies could be compared across disciplines to help policy makers decide in which new diagnostic technologies to invest.

### The value of the diagnostic randomized controlled trial design

In our example, the therapeutic response to the diagnostic question is whether to anticoagulate patients with confirmed DVT to prevent recurrent DVT. In the study by Kearon and colleagues [1] outcome event rates were not different, likely by virtue of missing clinically unimportant DVT in the serial ultrasound approach.

Venography is considered the reference standard for DVT. Diagnostic accuracy cohort studies would conclude that nothing could beat the “gold standard”. However, one could imagine a new test that does not diagnose clinically insignificant DVT (in other words, more specific for clinically relevant DVT) but identifies small DVT that venography might miss that will grow and ultimately become clinically significant. For Star Trek fans we will call this the “important clot tri-corder”. If the tri-corder were compared to venography in a diagnostic accuracy cohort study only, we might abandon the technology because of insensitivity for small inconsequential DVT. However, if a diagnostic randomized controlled trial were conducted, it is possible that we demonstrate its superiority to venography.

If venography were abolished by decree from government, we would not be left unable to evaluate diagnostic technologies for DVT. If we adopted diagnostic randomized controlled trials, the “important clot tri-corder” could be compared to serial ultrasounds and with clinical relevant important outcomes and then we could decide if the tri-corder should be adopted.

Venography is also complicated by the fact that it is frequently either indeterminate (inadequate contrast filling of veins) or cannot be done (unable to obtain venipuncture, contrast dye allergies, renal dysfunction). The 2 × 2 table and measures of diagnostic accuracy do not reflect this limitation of the test in actual practice nor the spectrum bias introduced by omitting these patients. The rate of indeterminate or “cannot be done tests” may be provided in manuscripts permitting the reader to contemplate the generalizability of a diagnostic cohort study to their setting but the rate of indeterminate or cannot be done tests is often omitted from publications of diagnostic cohort studies [9,10]. Diagnostic randomized controlled trials analyzed by “intention-to-test” would incorporate these often neglected outcome effects of either indeterminate tests or tests that could not be done in both arms of a trial. Furthermore, beyond generalizability, it is plausible that patients with indeterminate tests or tests that could not be done are different than those with determinate tests, and that these differences may confound the accuracy of a diagnostic test (in other words, spectrum bias). For example, venograms often cannot be done in patients with a lot of edema due to the inability to obtain a venipuncture for the procedure. However edema is a sign of important venous thrombosis (caused by large vein obstruction; for example, thigh veins). In a diagnostic cohort study, these patients would be excluded from the accuracy analysis and this will lead to a bias against the experimental diagnostic test. The important “clot tri-corder” would have made the diagnosis but given that fewer patients with thigh vein thrombosis relative to calf vein thrombosis (where there is usually less edema) would be included in the diagnostic accuracy analysis versus venogram, the tri-corder would seemingly have lower sensitivity. A diagnostic randomized controlled trial intention-to-test analysis would eliminate this bias.

Diagnostic accuracy can still be determined in a diagnostic randomized controlled trial when the gold standard and the experimental diagnostic test are conducted but the results of one test are randomly kept blinded (Figure4). In our example, both the tri-corder and venography could be conducted by the radiologist and only the result of the randomly assigned test are disclosed to the treating physician. Patient outcomes are then evaluated on follow-up. Diagnostic accuracy (sensitivity, specificity, likelihood ratios, accuracy etc.) of the tri-corder could still be reported at the conclusion of the trial in comparison to venography.

## Discussion

If the minister of health were trying to decide whether to invest in the tri-corder for DVT diagnosis or positron emission tomography (PET) scanning for early detection of lung cancer in patients with a lung mass, they could not make an informed decision based on diagnostic accuracy studies as these are only indirectly related to clinically relevant outcomes (mortality, surrogates of mortality, quality of life). However, if they had access to two diagnostic randomized controlled trials with clinically relevant outcomes, they could make a more informed decision on which technology to invest in (for example, one study comparing the tri-corder to venography with mortality as the outcome and a second study comparing computed tomography chest scanning to PET scanning for investigation of a lung mass with mortality as the outcome).

Diagnostic randomized controlled trials also eliminate or reduce the likelihood for many of the potential biases that threaten internal validity of diagnostic accuracy cohort studies (Table1). Randomization ensures that differential context bias is eliminated as both competing strategies would be evaluated in groups with similar disease prevalence. Clinicians often make between-study comparisons in interpreting the literature on diagnostic accuracy, increasing the likelihood of misinterpretation due to influence of disease prevalence on diagnostic accuracy measures (for example, predictive values). For example, ultrasound for DVT has much higher negative predictive values in low-risk community studies compared to high-risk inpatient studies. Conducting diagnostic randomized controlled trials of competing diagnostic strategies also reduces the risk of false conclusions from selection bias (patient populations with varying risks of false diagnosis); if adequate numbers of patients are randomized (usually >100) we ensure that the groups are balanced. For example, after a DVT there is often incomplete resolution leading to residual ultrasound abnormalities (that are not fresh dangerous clots but hard, often walled off, scars that are not at risk of embolizing). Randomization would ensure that these patients are balanced between the “tri-corder” group and venogram group and not drive the findings of our studies. On the other hand, a diagnostic accuracy study comparing the “tri-corder” to the venogram with a high prevalence of patients with prior DVT may conclude that the tri-corder missed a lot of DVTs (which were in fact old); in other words, conclude low sensitivity and low negative predictive values. Furthermore, examining an identical study with no patients with prior DVT would lead to higher sensitivity and negative predictive values. Differential clinical review is less likely to occur in a diagnostic randomized controlled trial as patient characteristics should be balanced in the different arms of a diagnostic randomized controlled trial. Test review and reference review bias are unlikely to occur with a diagnostic randomized controlled trial as the results are acted upon independently of the results of the alternative test. In diagnostic accuracy cohort studies, where both tests are conducted on a single patient, the tests are not necessarily independent of each other. An example is that venography has been thought to perhaps cause DVT. If the tri-corder were performed the next day after a negative venography and detected a DVT we would conclude that the tri-corder has identified a false positive. This would not occur in the diagnostic cohort study and, furthermore, would allow us to identify harm caused by diagnostic tests by having a reference group to compare incidences of adverse events.

**Table 1:** Threats to validity of diagnostic accuracy studies

**Common threats to validity**	**Definition**
**Internal validity (bias)**	
Context bias	Experimental test more likely to be reported as abnormal in populations with high disease prevalence
Clinical review bias	Experimental test or reference standard interpreted with knowledge of participant clinical characteristics
Test review bias	Experimental test interpreted with knowledge of the reference standard test results
Diagnostic review bias	Reference standard test interpreted with knowledge of the experimental test results
**External validity (generalizability)**	
Spectrum bias	Disease severity, participant demographics or participant co-morbidity influence experimental test accuracy
Limited challenge bias	Potential study participants with confounders known to influence experimental test accuracy excluded from study

Bossuyt and colleagues [11] have stated that randomized trials of diagnostic procedures offer several advantages over other design options, but they raise the important issue of efficiency. Specifically, randomizing patients who test positive to both diagnostic procedures or negative to both procedures may be inefficient as they do not contribute to the between-diagnostic comparison and their outcomes would be determined solely by the treatment not the test. Thus, only randomizing discordant patients (in other words, positive to one and negative to the other) to treatment arms would lead to better study efficiency. They offer an alternative design in which a cohort of patients would receive both diagnostic procedures and those patients with discordant findings would be randomized to different treatment strategies. However, the status of discordance would be known to treating staff at the time of randomization which could influence participation in the trial or treatment choices when randomized based on ambiguity of diagnosis. In addition, trying to consent patients and treating staff with known discordant status will likely further hamper recruitment. Second, discordant patients may have different demographic and clinical characteristics than concordant patients which could influence subsequent outcome rates and thus results may not be as generalizable as a diagnostic randomized trial.

As with any randomized trial, investigators need to ensure there is clinical equipoise between the interventions. For diagnostic randomized controlled trials, patients cannot be placed in a position of being randomized to a known inferior diagnostic procedure for the sake of research. Investigators must substantiate clearly that the interventions are in diagnostic equipoise.

While randomized trials are the gold standard for establishing effectiveness, they are generally more expensive and resource intensive than traditional research methods. Given the routine use of diagnostic procedures and their costs, we maintain that sacrificing validity of results with suboptimal designs is not prudent. Randomized controlled trials remain the standard study design for the approval of drugs and we feel the evaluation of diagnostic procedures should be treated no differently.

## Conclusion

Given the inherent limitations of diagnostic cohort studies, we suggest a greater awareness and uptake in the conduct of diagnostic randomized controlled trials. Doing so will better ensure that patients are offered effective diagnostic procedures that will make a clinical difference. The evaluation of diagnostic tests should be treated no differently than other interventions. The paucity of published diagnostic randomized controlled trials suggests we have a long way to go.

## Abbreviations

DVT: deep vein thrombosis; PET: positron emission tomography.

## Competing interests

The authors declare that they have no competing interests.

## Authors’ contributions

DF and MR conceived of the commentary and drafted the manuscript. All authors read and approved the final manuscript.
